# Notification that new names of prokaryotes, new combinations, and new taxonomic opinions have appeared in volume 73, part 12 of the IJSEM

**DOI:** 10.1099/ijsem.0.006243

**Published:** 2024-03-28

**Authors:** Aharon Oren, Markus Göker

**Affiliations:** 1 The Institute of Life Sciences, The Hebrew University of Jerusalem, The Edmond J. Safra Campus, 9190401 Jerusalem, Israel; 2 Leibniz Institute DSMZ – German Collection of Microorganisms and Cell Cultures, Inhoffenstrasse 7B, 38124 Braunschweig, Germany

This listing of names of prokaryotes published in a previous issue of the International Journal of Systematic and Evolutionary Microbiology (IJSEM) is provided as a service to microbiology to assist in the recognition of new names and new combinations. This procedure was proposed by the Judicial Commission [1]. The names given herein are listed according to the Rules of priority (i.e., time and date of publication of the articles on the journal’s platform and the order of valid publication of names in the original articles) [2].

**Table:** 

Order of article publication	Name/authors	Proposed as	Article no.	Page no.
1	*Thermococcus thermotolerans* Yang *et al*. 2023	sp. nov.	005934	8
2	*Pseudomonas aestuarii* Kim *et al*. 2023	sp. nov.	006190	5
3	*Pseudodonghicola flavimaris* Huang *et al*. 2023	sp. nov.	006192	7
3	*Sedimentitalea xiamensis* Huang *et al*. 2023	sp. nov.	006192	8
4	*Variovorax durovernensis* Alcolea-Medina *et al*. 2023	sp. nov.	006184	6
5	*Streptomyces koelreuteriae* Fu *et al*. 2023	sp. nov.	006196	6
6	*Flavobacterium sedimenticola* Wu *et al*. 2023	sp. nov.	006180	6
7	*Chryseobacterium pyrolae* Wang *et al*. 2023	sp. nov.	006068	6
8	*Micromonospora parastrephiae* Razmilic *et al*. 2023	sp. nov.	006189	11
8	*Micromonospora tarensis* Razmilic *et al*. 2023	sp. nov.	006189	11
9	*Hymenobacter endophyticus* Zhang *et al*. 2023	sp. nov.	006197	4
10	*Lactiplantibacillus brownii* Heng *et al*. 2023	sp. nov.	006194	9
11	*Marivirga aurantiaca* Zhang *et al*. 2023	sp. nov.	006195	7
12	*Halothiobacillus diazotrophicus* Dai *et al*. 2023	sp. nov.	006202	5
13	*Pseudooceanicola spongiae* Zhang *et al*. 2023	nom. nov. [basonym: *Pseudopuniceibacterium antarcticum* Liao *et al*. 2021]*	006207	1
14	*Brevibacillus ruminantium* Kim *et al*. 2023	sp. nov.	006204	6
15	*Frankia nepalensis* Nouioui *et al*. 2023	sp. nov.	006199	9
16	*Antiquaquibacter* Toumi *et al*. 2023	gen. nov.	006205	5
16	*Antiquaquibacter oligotrophicus* Toumi *et al*. 2023	sp. nov.	006205	8
17	*Pseudomonas aphyarum* Testerman *et al*. 2023	sp. nov.	006201	4
17	*Pseudomonas idahonensis* Testerman *et al*. 2023	sp. nov.	006201	6
17	*Pseudomonas rubra* Testerman *et al*. 2023	sp. nov.	006201	6
17	*Pseudomonas fontis* Testerman *et al*. 2023	sp. nov.	006201	7
18	*Tianweitania populi* (Li *et al*. 2016) Song *et al*. 2023	comb. nov. [basonym: *Corticibacterium populi* Li *et al*. 2016]	006193	6
18	*Tianweitania aestuarii* Song *et al*. 2023	sp. nov.	006193	6
19	*Marinomonas transparens* Cui *et al*. 2023	sp. nov.	005879	7
19	*Marinomonas sargassi* Cui *et al*. 2023	sp. nov.	005879	7
20	*Phytohabitans aurantiacus* Triningsih *et al*. 2023	sp. nov.	006106	8
21	*Tenacibaculum tangerinum* Lee *et al*. 2023	sp. nov.	006203	6
22	*Pseudomonas cucumis* Liao *et al*. 2023	sp. nov.	006208	8

*Illegitimate homotypic synonym: *Pseudooceanicola antarcticus* (Liao *et al*. 2021) Zhang *et al.* 2023.

The following taxonomic opinion (i.e., the emendation of a circumscription) does not affect the status of a name under the International Code of Nomenclature of Prokaryotes. This taxonomic opinion can neither be considered as approved nor as disapproved by the International Committee on Systematics of Prokaryotes.

**Table:** 

Name/authors:	Article no.	Page no.
*Tianweitania* Han *et al*. 2016 emend. Song *et al*. 2023	006193	7
